# Severe Fever With Thrombocytopenia Syndrome in Southeastern China, 2011–2019

**DOI:** 10.3389/fpubh.2021.803660

**Published:** 2022-02-09

**Authors:** Mingyong Tao, Ying Liu, Feng Ling, Yijuan Chen, Rong Zhang, Jiangping Ren, Xuguang Shi, Song Guo, Ye Lu, Jimin Sun, Jianmin Jiang

**Affiliations:** ^1^Medical School, Ningbo University, Ningbo, China; ^2^Key Laboratory of Vaccine, Prevention and Control of Infectious Disease of Zhejiang Province, Zhejiang Provincial Center for Disease Control and Prevention, Hangzhou, China

**Keywords:** severe fever with thrombocytopenia syndrome (SFTS), epidemiology, characteristic, spatiotemporal pattern analysis, tick

## Abstract

**Introduction:**

Severe fever with thrombocytopenia syndrome (SFTS) is an emerging infectious disease, and the number of cases has increased in recent years in Zhejiang Province, China. However, whether the seasonal distribution, geographic distribution, and demographic characteristics of SFTS have changed with the increase of incidence was unclear.

**Materials and Methods:**

Data on SFTS cases in Zhejiang Province and tick density in Daishan County from 2011 to 2019 were collected. The changing epidemiological characteristics of SFTS including seasonal distribution, geographical distribution, and demographic features were analyzed using descriptive statistical methods, Global Moran's I, local Getis-Ord *G*_i_* statistic, and spatial scan statistic.

**Results:**

A total of 463 SFTS cases including 53 (11.45%) deaths were reported from 2011 to 2019 in Zhejiang Province, and the annual number of cases showed increasing tendency. SFTS cases were reported in almost half of the counties (40/89) of Zhejiang Province. Elderly farmers accounted for most cases and the proportion of farmers has increased. Most cases (81.21%) occurred during April and August. The interval from illness onset to confirmation was significantly shortened (*Z* = 5.194, *p* < 0.001). The majority of cases were reported in Zhoushan City from 2011 to 2016, but most cases were reported in Taizhou City since 2017.

**Discussion:**

We observed dynamic changes in the seasonal distribution, geographical distribution, and demographic features of SFTS, and comprehensive intervention measures, such as clearance of breeding sites, killing of tick adults, and health education should be strengthened in farmers of the key areas according to the changed epidemiological characteristics.

## Introduction

Severe fever with thrombocytopenia syndrome (SFTS), caused by SFTS virus (SFTSV), was firstly confirmed in China in 2009 (1). SFTSV belongs to genus Phlebovirus in family Phenuiviridae, order Bunyavirales. In 2009, SFTS cases were firstly reported in Hubei Province and Henan Province, China. Subsequently, SFTS cases were also identified in South Korea, Japan, United Arab Emirates, and Vietnam (2–5). In 2014, a new phlebovirus associated with severe febrile illness was identified in Missouri (6). Moreover, Zwiesel bat banyangvirus, a potentially zoonotic SFTSV-like banyangvirus was detectable in Northern bats from Germany (7). SFTSV has a wide range of vertebrate hosts, including domestic and wild animals, such as goats, sheep, cattle, dogs, pigs, and chickens (8, 9). Of note, direct infection of SFTSV from cats and dogs to humans has been reported and recognized as a social problem (10–13).

The main clinical manifestations of SFTS included high fever, gastrointestinal symptoms, and bleeding tendency. Most SFTS patients went through a self-limiting clinical course, but some cases developed serious symptoms (8, 14–17). Some of the patients (12–30%) may develop thrombocytopenia and leukopenia, and then die due to multiple organ failure (9, 14, 17–19). WHO had listed SFTS as one of the nine infectious diseases on its priority list, due to the increasing number of SFTS cases and its potential threat to human health.

Since 2010, more than 5,000 SFTS cases have been reported in more than 15 provinces of China (15). According to a survey of seroprevalence of SFTSV antibodies in Zhejiang Province, SFTS was endemic in rural areas of Zhejiang Province, and more than 400 SFTS cases have been reported since 2011 (20). With the development of detection technology, increasing awareness, and the change of environment factors, more and more SFTS cases were identified in Zhejiang Province. Here, we aim to summarize the changing epidemiological characteristics of SFTS in Zhejiang Province and analyze whether some characteristics have changed which would provide scientific information for its accurate control and prevention.

## Materials and Methods

### Case Definition

A person with acute onset of fever (≥38.0°C) and other symptoms (e.g. gastrointestinal symptoms, bleeding), epidemiological risk factors (being a farmer or being exposed to ticks two weeks before illness onset) and laboratory data consisting of thrombocytopenia and leukocytopenia was defined as a suspected case. A suspected case with one or more of the following criteria: (1) detection of SFTSV RNA, (2) seroconversion or 4-fold increase in antibody titers between paired serum samples collected at least two weeks apart, and (3) isolation of SFTSV in cell culture was defined as a confirmed case.

### Data Collection

**Figure 1:**
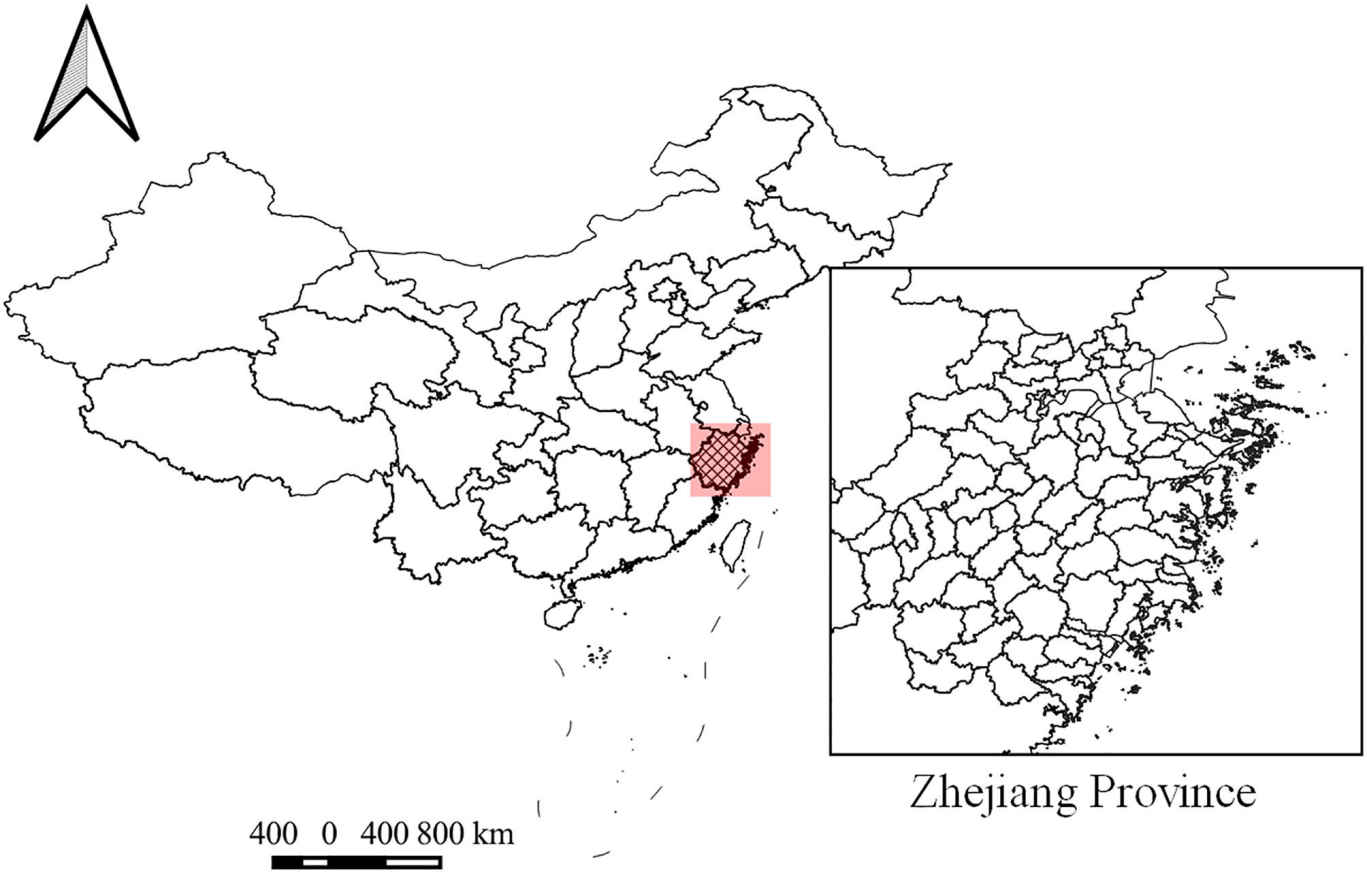
The location of Zhejiang Province.

Zhejiang Province, one of the southeastern coastal provinces of China, is located at 27°02'N to 31°11'N and 118°01'E to 123°10'E (Figure 1). The cities of Zhejiang Province and their subordinate counties are listed in Supplementary Table 1. The surveillance data of SFTS cases from January 2011 to December 2019 in Zhejiang Province were extracted from the communicable disease surveillance network system. We collected the following data: residential address, age, gender, occupation, date of illness onset, date of case confirmation, and the outcome (recovered or fatal). Furthermore, the demographic data of every county were collected from the statistical yearbook of Zhejiang Province.

### Ethics Statement

This present research reported here has been approved by the Ethics Committee of Zhejiang Provincial CDC. Human research was carried out in accordance with the provisions of the Declaration of Helsinki.

### Data Analysis

Means and *SD* ($\bar{x}$ ± *s*) were applied if data conformed to normal distribution, otherwise median with interquartile range (*M* [*Q*_1_, *Q*_3_]) were used to describe the distribution of age, gender, occupation, duration onset to diagnosis annually. Enumeration data were described with frequencies and proportions annually. The Kruskal–Wallis test was used to compare the distribution of duration from illness onset to confirmation and the age distribution of SFTS cases. The chi-square test was used to compare SFTS's distribution of gender and occupation. The Jonckheere–Terpstra test, the Mann–Kendall trend test, and linear-by-linear association were used to conduct the trend analysis. *ArcGis* 10.5 (ESRI, Redlands, CA, USA) was used to describe the geographical distribution of SFTS cases, and basic analysis was performed using *R* software version 4.0.0 (R Foundation for Statistical Computing, Vienna, Austria) and SPSS 18.0 (IBM Corp., Armonk, NY, USA).

### Spatial Autocorrelation Analysis

Global Moran's *I* was used to explore the spatial pattern of the whole study area, the range of Global Moran's *I* is [−1,1], and how closer its value is to 1. The closer the relationship between the spatial units is, the more similar their properties are, conversely, the closer it is to −1, the greater the difference between the spatial units (21). *Z* statistics and values were according to the results of Monte Carlo tests (999 times), and the main spatial weight matrix was set by the Queen method.

In addition, spatial heterogeneity is defined as the presence of significant variation in spatial autocorrelation within the study area. It is necessary to describe the variation of spatial autocorrelation in the study area. Therefore, we must rely on the local indexes that can detect local spatial autocorrelation to provide spatial autocorrelation values of regional units. Local indicators of spatial association were depicted by Local Getis-Ord *G*_i_* statistic (22, 23). The expected value and the variance of the Local Getis-Ord *G*_i_* statistic could be calculated by computer, so as to conduct the hypothesis test of whether the *G*_i_* of the population is zero and deduce whether there are statistically significant high-value or low-value aggregation areas in the region and adjacent areas where the disease space *i* and *j* is distributed.

There are three categories on the *G*_i_* Cluster Map: High-High (hot spots); Low-Low (cold spots); None (spatial outliers not significant). Spatial autocorrelation analysis was performed using *GeoDa* version 1.14.0.

### Space-Time Cluster Analysis

Spatial scan statistics was initially used to monitor the clusters of temporal aggregation. Additionally, the analysis was extended to three-dimensional space-time cluster by Kulldorff, which had been widely used in epidemiology and other fields. A cylindrical scanning window is established in the counties area of the research, and the bottom of the cylinder represents the size of the scanning area, and the height of the cylinder represents the length of the time range. The risk in each scanning window was examined, and the likelihood ratio (LLR) was also calculated. The value of LLR in different windows could be calculated and the value could be considered as statistics to compare and test by Monte-Carlo sampling. In our study, discrete Poisson distribution was selected to fit the probability model, high rates were set to scan for areas, the maximum spatial cluster size was reconfigured as 25% of the population at risk in the spatial window, the maximum temporal cluster size was given 10% of the study period (12 months), and the maximum number of replications was set as 999. The space-time cluster analysis was programmed in Software for the Spatial and Space-Time Scan Statistic (SaTScan version 9.3), and the result was performed using *ArcGis* 10.5 (ESRI, Redlands, CA, USA).

### The Temporal and Spatial Distribution of Ticks in Daishan County

Daishan County is one of the most SFTS affected areas in Zhejiang Province, where ticks monitoring site was set up in 2013. There were three monitoring sites before 2017 and five monitoring sites after 2017 in Daishan County.

## Results

### Epidemiological Characteristics

**Figure 2:**
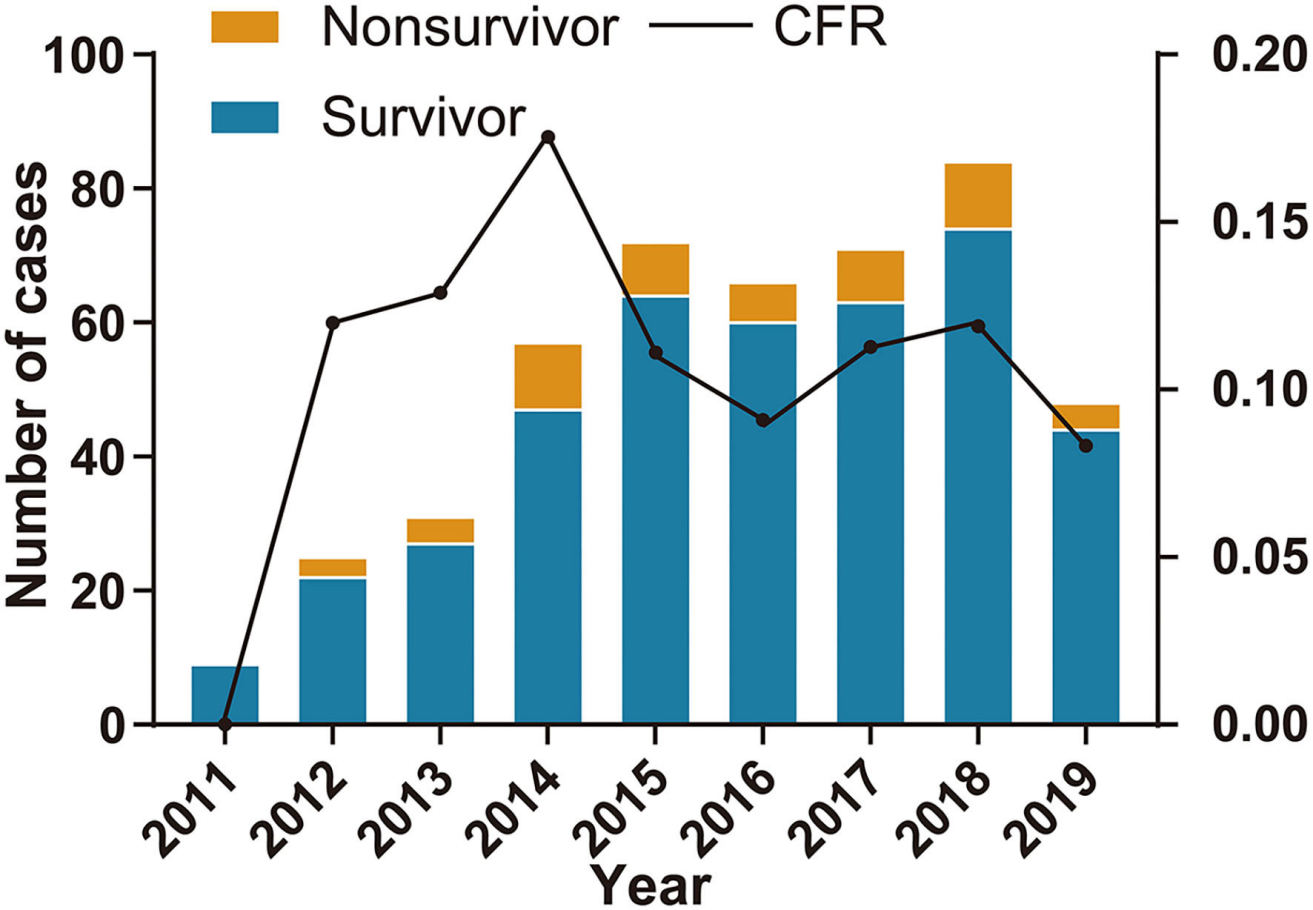
The number of severe fever with thrombocytopenia syndrome (SFTS) cases and case fatality rate in Zhejiang Province from 2011 to 2019.

A total of 463 confirmed SFTS cases were reported from 2011 to 2019 in Zhejiang Province. The annual case was 9, 25, 31, 57, 72, 66, 71, 84, and 48 from 2011 to 2019 which showed an increasing tendency (Figure 2). The average case fatality rate (CFR) was 11.45% (53/463) and the difference of CFR in different years was not significant.

Of the 463 cases, 223 were men and 240 were women (the male-to-female ratio was about 0.929), and there was a significant difference (χ^2^ = 15.192, *p* = 0.034) across the different years. However, we found that the male-to-female ratio (ratio = 0.44) was particularly low in 2015, and the annual male-to-female ratio did not differ significantly (χ^2^ = 4.601, *p* = 0.596) across the other different years (Supplementary Table 2).

**Figure 3:**
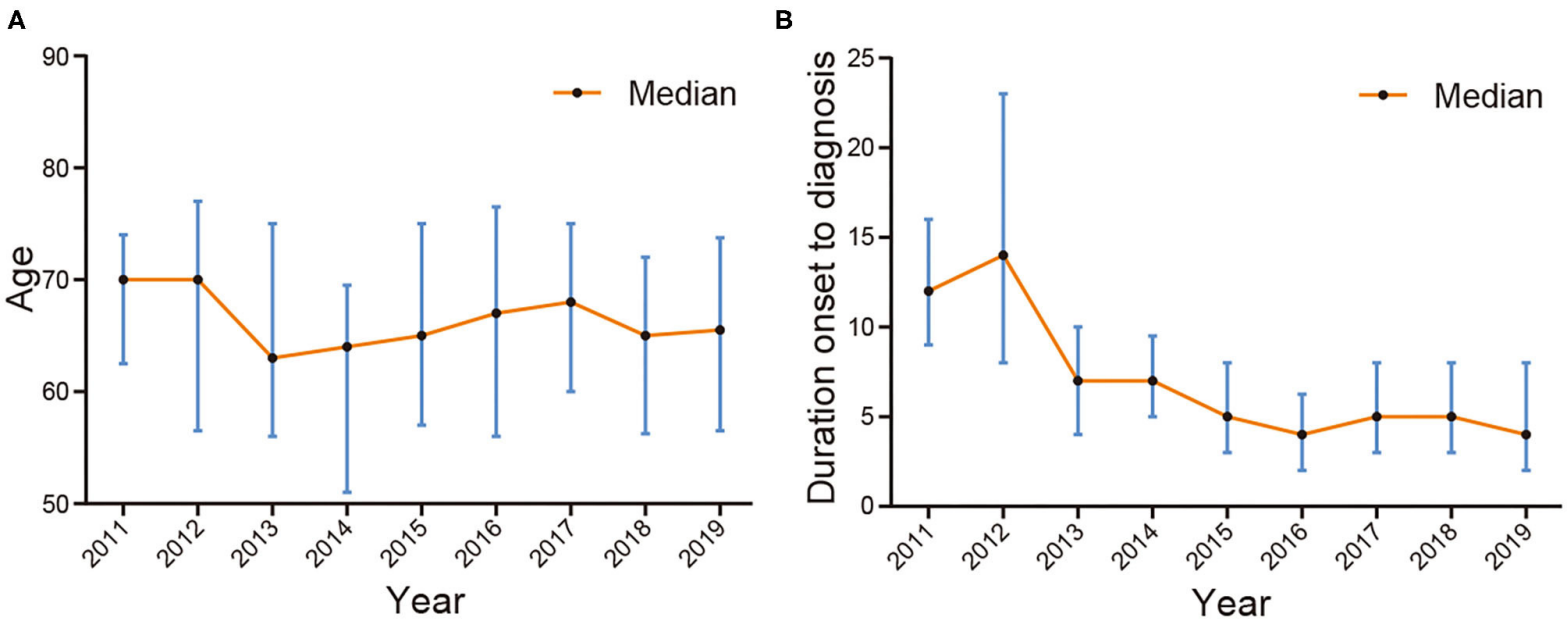
The median age and interval from illness onset to confirmation of SFTS cases in Zhejiang Province from 2011 to 2019. **(A)** is the median age and **(B)** is the interval from illness onset to confirmation.

The median age of all 463 cases was 66.00 (57.00, 74.00) and the annual median ages of cases from 2011 to 2019 were 70.00, 70.00, 63.00, 64.00, 65.00, 67.00, 68.00, 65.00, and 65.50, respectively (Figure 3A). The age distribution of different years was similar (χ^2^ = 10.167, *p* = 0.254). Of 463 SFTS cases, 321 cases (69.33%) were farmers accounting for the largest proportion and the proportion of farmers increased from 2011 to 2019 (*p* < 0.001).

**Figure 4:**
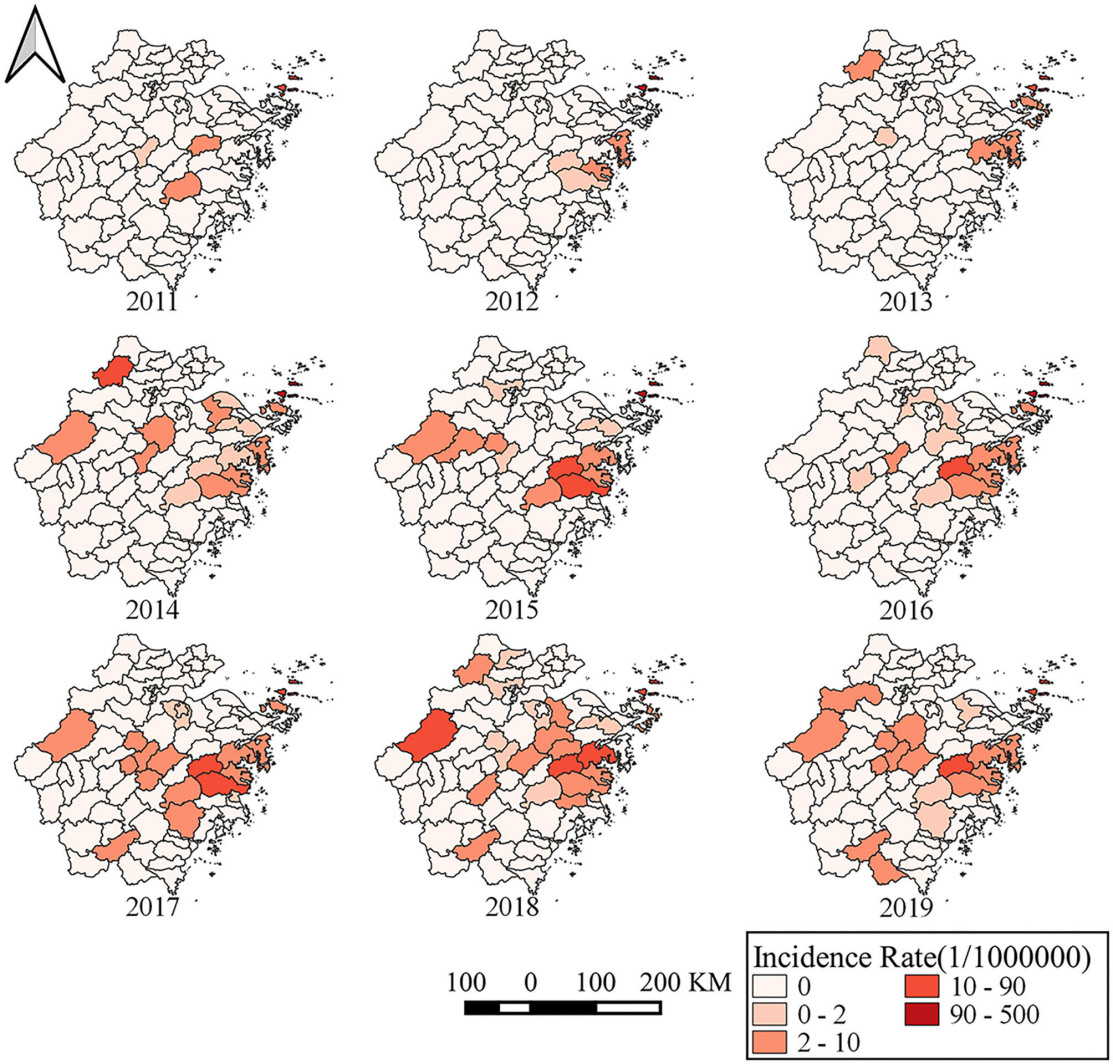
The annual incidence rates of different counties in Zhejiang Province from 2011 to 2019.

From 2011 to 2019, SFTS cases were identified in 40 counties of Zhejiang Province. Of the 463 SFTS cases, 133 cases were reported in Daishan County, 71 cases in Linhai County, 51 cases in Tiantai County, 25 cases in Ninghai County, and 20 cases in Chun'an County. The numbers of SFTS affected counties from 2011 to 2019 were 4, 5, 7, 15, 13, 15, 20, 24, and 19, respectively (Figure 4).

**Figure 5:**
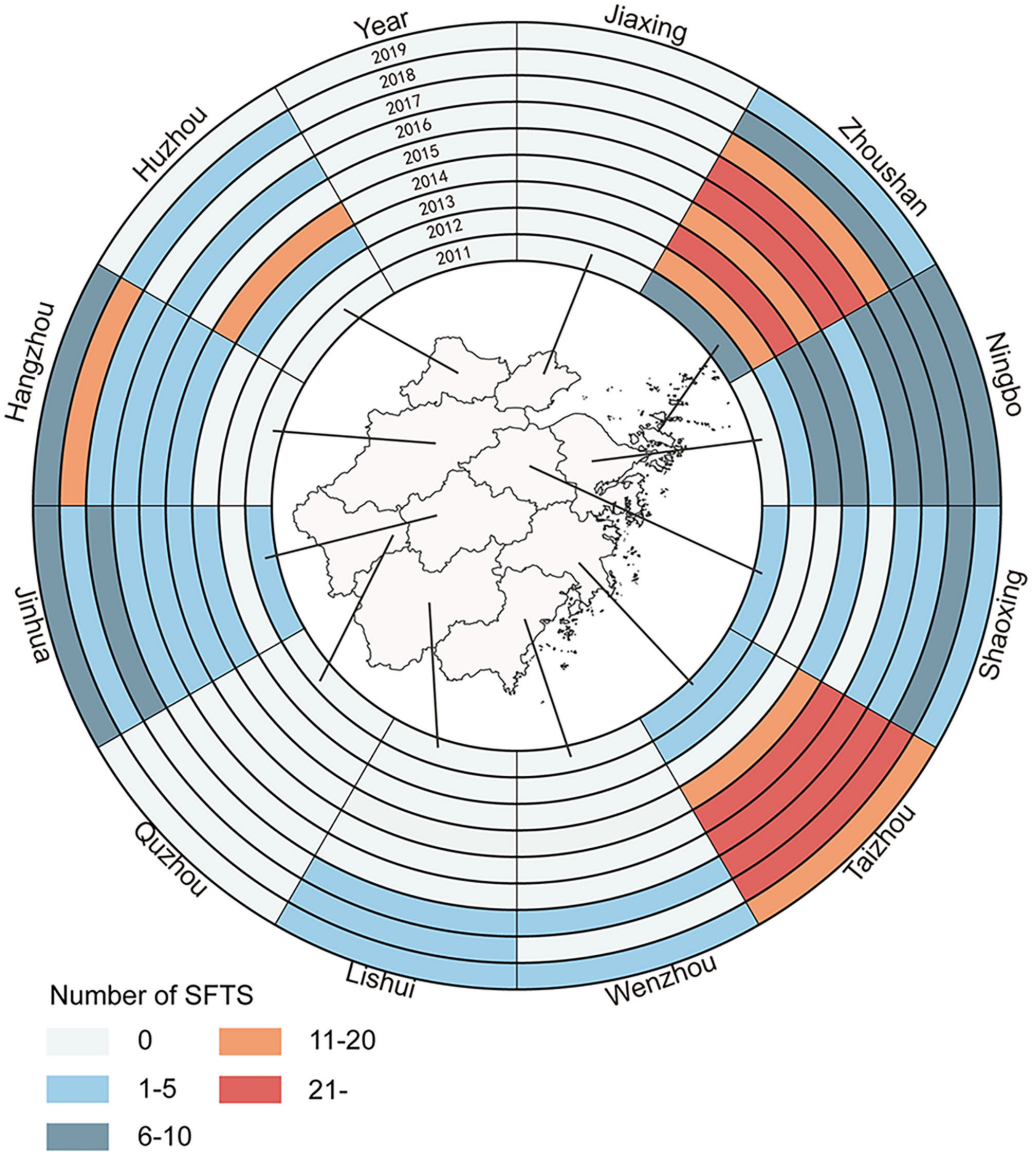
Spatio-temporal distribution of SFTS cases in Zhejiang Province from 2011 to 2019.

The numbers of cases reported in Taizhou City, Zhoushan City, Ningbo City, Jinhua City, Hangzhou City, Shaoxing City, Huzhou City, Wenzhou City, and Lishui City were 158, 147, 54, 37, 27, 18, 15, 4, and 3, respectively. Notably, the numbers of SFTS cases were 6, 18, 21, 19, 31, 26, 14, 9, and 3 in Zhoushan City during 2011–2019, but the corresponding numbers were 1, 3, 0, 11, 32, 25, 33, 34, and 19 in Taizhou City. About 48.97% (95/194) of SFTS cases were reported in Zhoushan City from 2011 to 2015, but 41.26% (111/269) of the cases were reported in Taizhou City from 2016 to 2019 (Figure 5). The most seriously affected areas shifted from Zhoushan City to Taizhou City.

**Figure 6:**
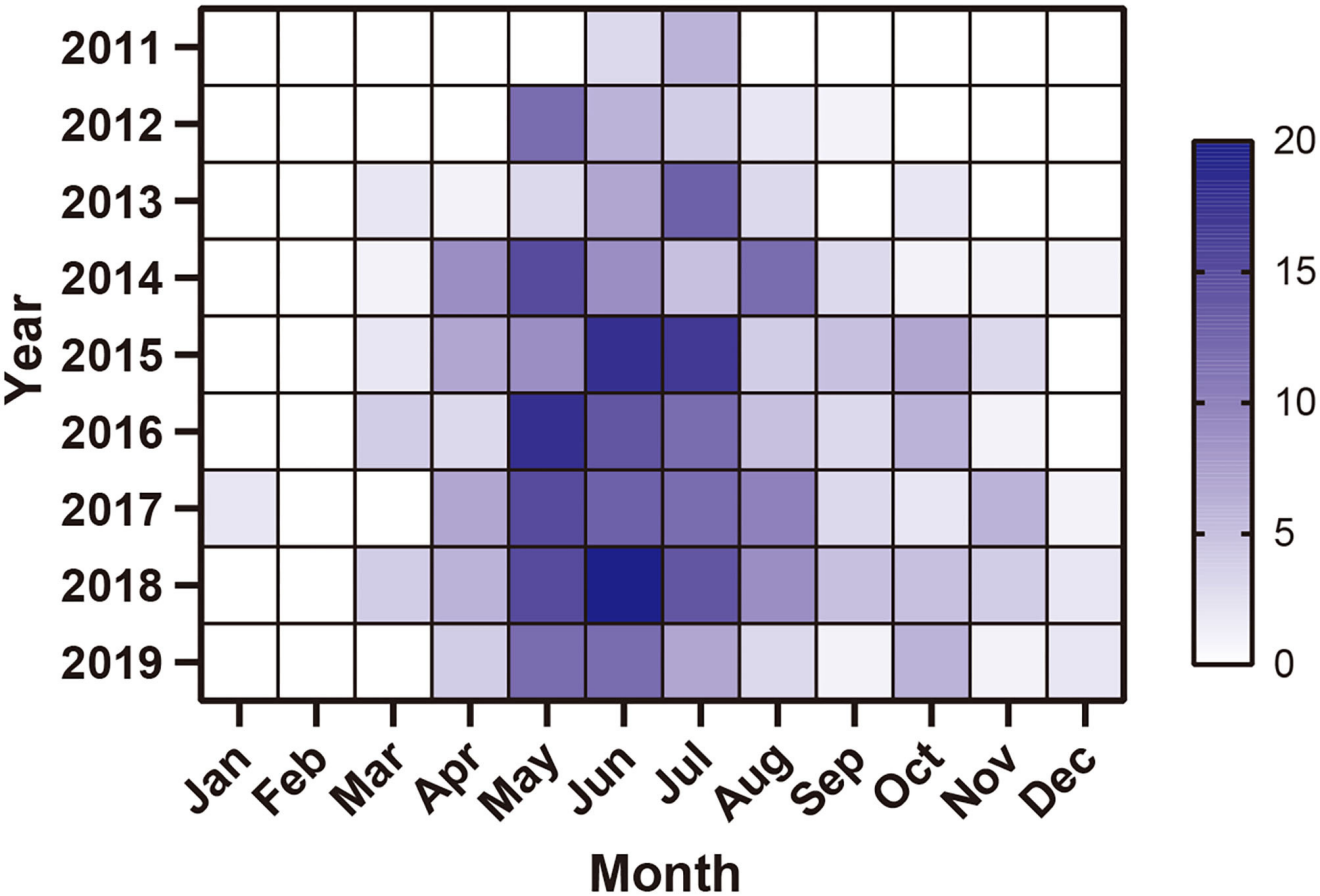
Monthly distribution of SFTS cases in Zhejiang Province from 2011 to 2019.

The heat map showed the monthly distribution of SFTS cases in Zhejiang Province from 2011 to 2019. A total of 376 cases (81.21%) occurred during April and August, and 426 cases (92.01%) occurred during April and October (Figure 6).

The median interval from illness onset to diagnosis of all cases was 6.00 (3.00, 9.00) days and the intervals from 2011 to 2019 were 12.00, 14.00, 7.00, 7.00, 5.00, 4.00, 5.00, 5.00, and 4.00 days, respectively. Of note, there was a significant difference (Kruskal–Wallis Test χ^2^ = 73.911, *p* < 0.001) between the intervals in different years and a downtrend from 2011 to 2019 (Figure 3B).

### Spatial Autocorrelation Analysis

**Figure 7:**
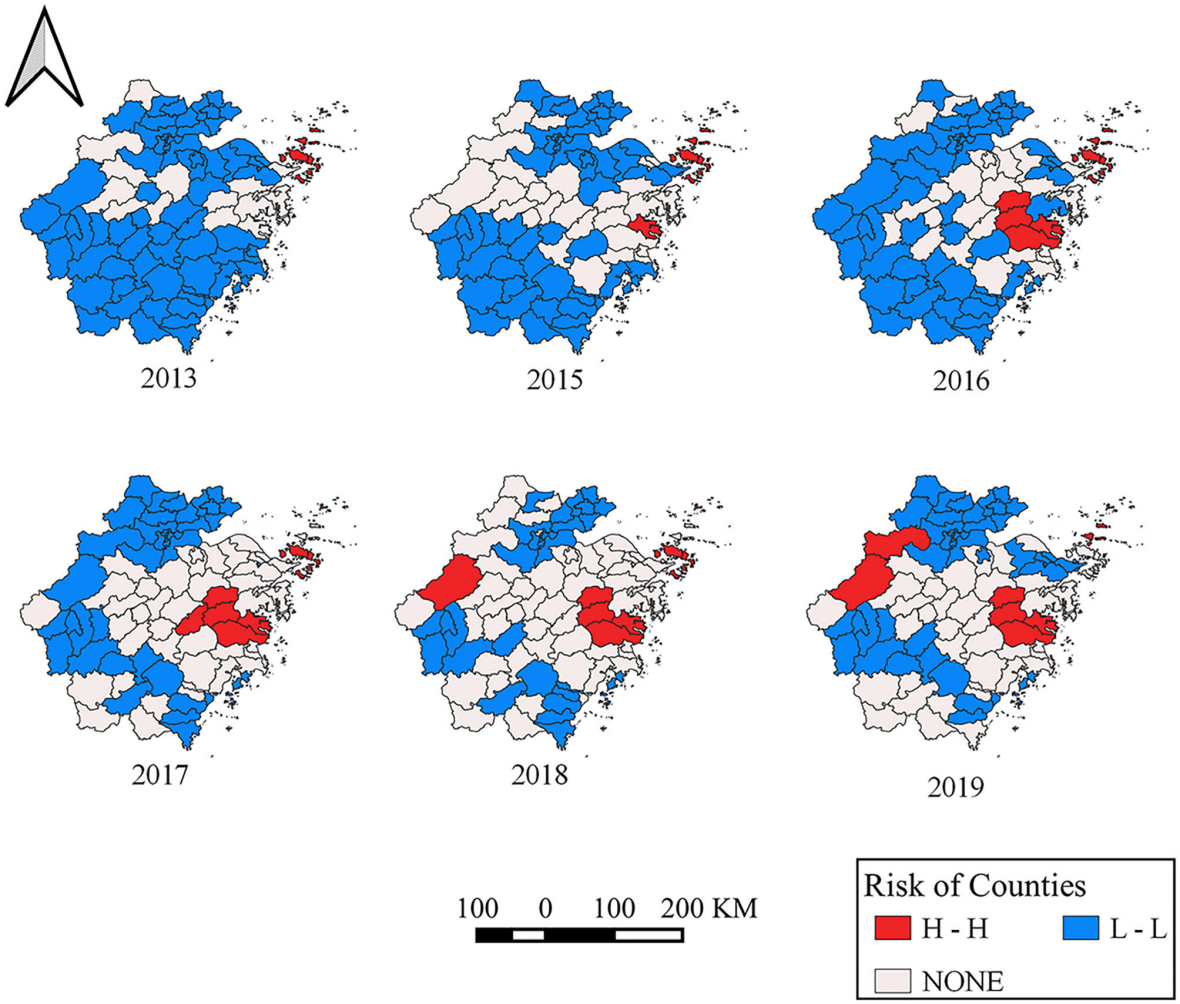
Annual local Getis-Ord *G*_i_*Cluster Map in Zhejiang Province from 2011 to 2019.

The Moran's *I* by global spatial autocorrelation analysis showed that positive global spatial autocorrelations (Moran's *I* = 0.491 in 2013, 0.431 in 2015, 0.401 in 2016, 0.513 in 2017, 0.187 in 2018, and 0.270 in 2019), which indicated that the positive correlation property of the regional distribution of SFTS cases (Table 1). The results of local spatial autocorrelation showed that the hot spot clusters (H–H, red color area) mainly located in some islands of southeastern coastal areas of Zhejiang Province, including Daishan, Shengsi, Dinghai, and Putuo, central-eastern areas of Zhejiang Province, including Tiantai, Ninghai, Linhai, Xiangshan, Sanmen, and Xianju from 2016, and western areas of Zhejiang Province, including Chun'an, Lin'an, and Anji from 2018 (Figure 7).

**Table 1:** The Moran's *I* of global spatial autocorrelation analysis during 2011–2019.

**Year**	**Moran's1** ***I***	***Z*** **score**	***P***-**value**
2011	−0.035	−0.407	0.295
2012	0.044	1.575	0.081
2013	0.491	8.860	0.015
2014	−0.014	−0.161	0.419
2015	0.431	7.358	0.003
2016	0.401	6.430	0.002
2017	0.513	7.860	0.001
2018	0.187	3.332	0.011
2019	0.270	4.262	0.002

### Space-Time Cluster Analysis

**Figure 8:**
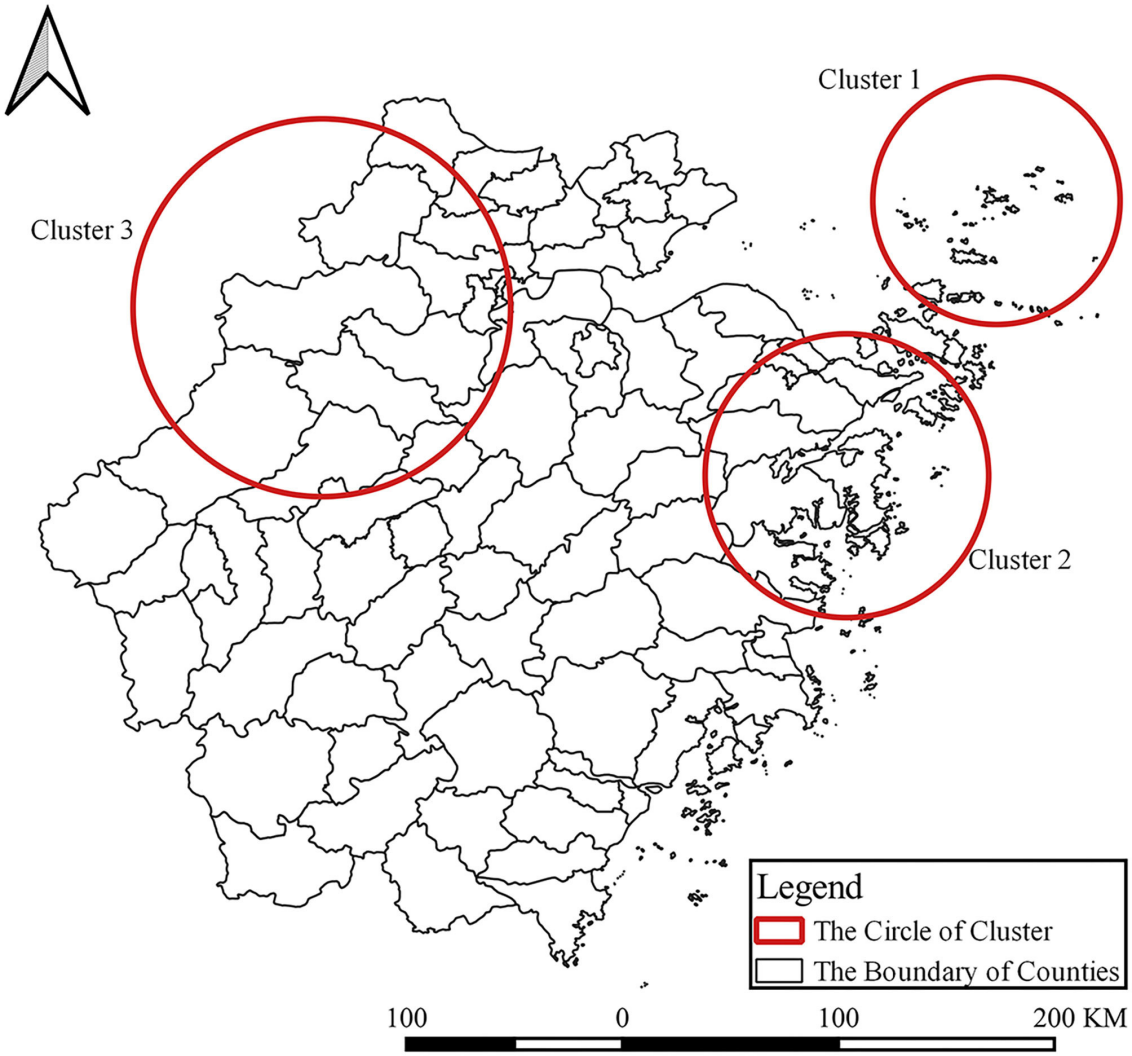
Time-space clusters of SFTS cases at county level in Zhejiang Province from 2011 to 2019.

As summarized in Table 2, the most likely cluster mainly distributed in Zhoushan City (LLR = 29.228, *p* = 1.200E-11 < 0.001) including Daishan County, Shengsi County from 2015/4/27 to 2015/10/23. The secondary likely clusters are located in the east coast and northwest areas of Zhejiang Province. One cluster, including Xiangshan County, Ninghai County, Sanmen County, Tiantai County, Yinzhou County, Haishu County, Putuo County, Dinghai County (LLR = 8.037, *p* = 0.021) from 2017/4/16 to 2017/10/12, and the relative risk (RR) was 3.72. Additionally, another spatial-temporal cluster including Lin'an County, Anji County, Chun'an County, Zhuji County, Yuhang County, Jiande County, Pujiang County, Changxing County, Wuxing County, and Xiaoshan County from 2014/5/2 to 2014/10/28, and RR was 4.19. The other cluster included Lin'an County, Anji County, Chun'an County, Zhuji County, Yuhang County, Jiande County, Pujiang County, Changxing County, Wuxing County, and Xiaoshan County, RR was 4.19, the time frame of Cluster 3 was from 2014/5/2 to 2014/10/28 (Figure 8).

**Table 2:** The results of spatial-temporal scanning.

**Clusters**	**Principal**	**Secondary**	
	**Cluster 1**	**Cluster 2**	**Cluster 3**
Coordinates	30.71 N, 122.48 E	29.38 N, 121.85 E	30.21 N, 119.34 E
Radius	48.01 km	78.85 km	106.00 km
Time frame	2015/4/27-2015/10/23	2017/4/16-2017/10/12	2014/5/2-2014/10/28
Location counties	Daishan, Shengsi	Xiangshan, Ninghai, Sanmen	Lin'an, Anji, Chun'an, Zhuji
		Tiantai, Yinzhou, Haishu	Yuhang, Jiande, Pujiang
		Putuo, Dinghai	Changxing, Wuxing, Xiaoshan
No. of cases	27.00	14.00	12.00
Expected	3.99	3.85	2.93
Annual cases (1/100,000)	60.80	32.60	36.80
O/E	6.77	3.64	4.10
Relative risk	7.13	3.72	4.19
LLR	29.228	8.037	7.954
*P*-value	1.200E-11	0.021	0.025

### The Temporal and Spatial Distribution of Ticks in Daishan County

Before 2017, the density of ticks ranged from 12.00 to 20.00. However, the density ranged from 9.20 to 12.00 in 2017, 2.20 to 4.13 in 2018, and 1.2 to 4.13 in 2019. The tick density was apparently on a downward trend.

## Discussion

In this study, we investigated the changing epidemiological characteristics of SFTS cases in Zhejiang Province from 2011 to 2019. Our study confirmed that there was an increasing tendency in the number of annual SFTS cases and affected counties. Several factors may be associated with the results. Since the identification of SFTS, more attention has been paid by health departments, and some measures have been conducted to improve the capacity of diagnosis in Zhejiang Province. For example, all municipal cities and counties where more than 5 cases had been reported established detection methods for SFTSV in Zhejiang Province in 2014 (8, 15, 16, 24). The improvement of diagnosis and detection capacity may increase the number of reported SFTS cases. Similarly, the interval from illness onset to confirmation was significantly shortened from 2011 to 2019.

We also found that the medians of SFTS cases were all >60 years from 2011 to 2019, and the majority of the cases were farmers. The previous studies had reported that the elderly individuals might be more likely infected by SFTSV due to a decrease in immunity and underlying medical conditions (15, 16, 24). Another factor may also contribute to the result. In these affected counties, the majority of cases were elderly farmers living in hilly areas as young people went to cities to earn better money. Therefore, the elderly farmers had more chance of exposure to tick.

Similar to the results of other studies, most SFTS cases occurred from April to October. The seasonal distribution of SFTS cases might be related to tick dynamics. Ticks were believed as the carriers of SFTSV, and *Haemaphysalis longicornis* was the predominant population carrying SFTSV (25, 26). The major habitats of *H. longicornis* were the shrub, grass, and broad-leaved forest habitat. *H. longicornis* were active in early March on vegetation, and there was an overlap among three stages with larvae active in late June. A number of *H. longicornis* achieved peak in September, with nymphs active in early April and reaching peak in May and adults active from March to September and reaching peak in July (27–29). Moreover, the heat map of the monthly distribution of SFTS cases indicated that SFTS cases concentrated from April to October. The ecological characteristic of *H. longicornis* was also in agreement with the corresponding results from the epidemiology of SFTS. Most ticks, especially those living in temperate and subtropical regions, had a distinct seasonal cycle in finding their hosts.

Of interest, most SFTS cases were reported in areas of Zhoushan City before 2016 and then the number of SFTS cases in Zhoushan City decreased sharply. The measures such as health education and risk communication, weed removal, livestock management, and tick control had been conducted since 2016 in Zhoushan City (24). Staff in Zhoushan City removed shrubs from residents' surroundings, cleaned the lane around the house, and eradicated the weeds on both sides of the channel. Villagers cleared a width of at least 1 m above the no turf isolation belt and cleaned up the free ticks of the household environment and the ticks attached to raising animals. Health cleaning and insecticidal was implemented once a month for domestic animals and livestock (15, 24). As a result, the number of SFTS cases and tick density decreased in Zhoushan City. Our study also indicated that tick density decreased sharply in Daishan County. Other factors such as the SFTSV-carrying rate among ticks and molecular epidemiological characteristics of SFTSV may also contribute to SFTS incidence. To our disappointment, SFTSV was not detectable among ticks from Zhoushan City.

We used the global Moran's *I* index to detect whether the positive spatial autocorrelation existed, and used Getis-Ord *G*_i_* to explore the high-risk hotspots. We found hotspots and the gathering center was expanding from 1 center to 3 centers. Moreover, high-risk areas are mainly located at Daishan—Shengsi, Ninghai—Sanmen—Xiangshan—Linhai, and Anji—Lin'an—Chun'an. The heat map of the monthly distribution of SFTS cases indicated that SFTS cases concentrated from April to October, which was accordant with the gathering time of three spatial-temporal clusters. Of note, the time frame of clusters indicated that the center of a cluster of SFTS was not only transferring but also expanding.

There are several limitations to the study. First, data of SFTS cases were collected from a passive surveillance system. Some factors such as detection capability and availability of health facilities may influence the data quality. Second, other factors may also be associated with SFTS incidence. For example, the SFTSV-carrying rate of ticks, molecular epidemiological characteristics of SFTSV, and the meteorological factor may be associated with SFTS occurrence. But we could not get data on these factors in the study, and additional research is warranted to elucidate the factors associated with SFTS incidence.

In summary, our study analyzed the changing epidemiological characteristics of SFTS in Zhejiang Province. We found that the annual number of SFTS cases and affected counties showed an increasing tendency in Zhejiang Province. Some epidemiological characteristics have changed. For example, high incidence areas transferred from Zhoushan City to Taizhou City, the proportion of farmers increased year by year, and the interval from illness onset to confirmation decreased year by year. Comprehensive intervention measures such as clearance of breeding sites, kill of tick adults, and health education should be adjusted according to the changed epidemiological characteristics.

## Data Availability Statement

The raw data supporting the conclusions of this article will be made available by the authors, without undue reservation.

## Author Contributions

MT is responsible for conceptualization, methodology, software, writing of the original draft, reviewing and editing. YL and FL are responsible for conceptualization, methodology, software, and data curation. YC is responsible for software and visualization. RZ, JR, XS, and SG are responsible for data curation and supervision. YL, JS, and JJ are responsible for conceptualization, methodology, software, data curation, writing, reviewing and editing. All authors contributed to the article and approved the submitted version.

## Funding

This study was supported by a grant from the science technology department of Zhejiang Province (No. LGF20H260001) and the state project for scientific and technological development of the 13th Five-Year plan in China (No. 2017ZX10303404008002).

## Conflict of Interest

The authors declare that the research was conducted in the absence of any commercial or financial relationships that could be construed as a potential conflict of interest.

## Publisher's Note

All claims expressed in this article are solely those of the authors and do not necessarily represent those of their affiliated organizations, or those of the publisher, the editors and the reviewers. Any product that may be evaluated in this article, or claim that may be made by its manufacturer, is not guaranteed or endorsed by the publisher.

## References

[1] Yu XJ Liang MF Zhang SY Liu Y Li JD Sun YL. Fever with thrombocytopenia associated with a novel bunyavirus in China. N Engl J Med. (2011) 364:1523–32. 10.1056/NEJMoa1010095 21410387 PMC3113718

[2] Denic S Janbeih J Nair S Conca W Tariq WUZ Al-Salam S. Acute thrombocytopenia, leucopenia, and multiorgan dysfunction: the first case of SFTS Bunyavirus outside China? Case Rep Infect Dis. (2011) 2011:204056. 10.1155/2011/204056 22567462 PMC3336226

[3] Kim KH Yi J Kim G Choi SJ Jun KI Kim NH. Severe fever with thrombocytopenia syndrome, South Korea, 2012. Emerg Infect Dis. (2013) 19:1892. 10.3201/eid1911.130792 24206586 PMC3837670

[4] Takahashi T Maeda K Suzuki T Ishido A Shigeoka T Tominaga T. The first identification and retrospective study of severe fever with thrombocytopenia syndrome in Japan. J Infect Dis. (2014) 209:816–27. 10.1093/infdis/jit603 24231186 PMC7107388

[5] Tran XC Yun Y Le Van An S.-H. K. Thao NTP Man PKC. Endemic severe fever with thrombocytopenia syndrome, Vietnam. Emerg Infect Dis. (2019) 25:1029. 10.3201/eid2505.181463 31002059 PMC6478219

[6] McMullan LK Folk SM Kelly AJ MacNeil A Goldsmith CS Metcalfe MG. A new phlebovirus associated with severe febrile illness in Missouri. N Engl J Med. (2012) 367:834–41. 10.1056/NEJMoa1203378 22931317

[7] Kohl C Brinkmann A Radonic A Dabrowski PW Nitsche A Muhldorfer K. Zwiesel bat banyangvirus, a potentially zoonotic Huaiyangshan banyangvirus (Formerly known as SFTS)-like banyangvirus in Northern bats from Germany. Sci Rep. (2020) 10:1370. 10.1038/s41598-020-58466-w 31992832 PMC6987236

[8] Chen C Li P Li KF Wang HL Dai YX Cheng X. Animals as amplification hosts in the spread of severe fever with thrombocytopenia syndrome virus: a systematic review and meta-analysis. Int J Infect Dis. (2019) 79:77–84. 10.1016/j.ijid.2018.11.017 30500443

[9] Han SW Kang JG Byeon AR Cho YK Choi KS Chae JS. Severe fever with thrombocytopenia syndrome in canines from the Republic of Korea. Ticks Tick Borne Dis. (2020) 11:101454. 10.1016/j.ttbdis.2020.101454 32370926

[10] Ando T Nabeshima T Inoue S Tun M Obata M Hu W. Severe fever with thrombocytopenia syndrome in cats and its prevalence among veterinarian staff members in Nagasaki, Japan. Viruses. (2021) 13:1142. 10.3390/v13061142 34198717 PMC8232257

[11] Kida K Matsuoka Y Shimoda T Matsuoka H Yamada H Saito T. A Case of cat-to-human transmission of severe fever with thrombocytopenia syndrome virus. Jpn J Infect Dis. (2019) 72:356–8. 10.7883/yoken.JJID.2018.526 31366857

[12] Park E Shimojima M Nagata N Ami Y Yoshikawa T Iwata-Yoshikawa N. Severe fever with thrombocytopenia syndrome phlebovirus causes lethal viral hemorrhagic fever in cats. Sci Rep. (2019) 9:11990. 10.1038/s41598-019-48317-8 31427690 PMC6700174

[13] Yamanaka A Kirino Y Fujimoto S Ueda N Himeji D Miura M. Direct transmission of severe fever with thrombocytopenia syndrome virus from domestic cat to veterinary personnel. Emerg Infect Dis. (2020) 26:2994. 10.3201/eid2612.191513 33219655 PMC7706950

[14] Sun J Lu L Wu H Yang J Liu K Liu Q. Spatiotemporal patterns of severe fever with thrombocytopenia syndrome in China, 2011-2016. Ticks Tick Borne Dis. (2018) 9:927–33. 10.1016/j.ttbdis.2018.03.026 29606619

[15] Sun J Lu L Wu H Yang J Ren J Liu Q. The changing epidemiological characteristics of severe fever with thrombocytopenia syndrome in China, 2011-2016. Sci Rep. (2017) 7:9236. 10.1038/s41598-017-08042-6 28835633 PMC5569157

[16] Sun J Chai C Lv H Lin J Wang C Chen E. Epidemiological characteristics of severe fever with thrombocytopenia syndrome in Zhejiang Province, China. Int J Infect Dis. (2014) 25:180–5. 10.1016/j.ijid.2014.02.022 24947422

[17] Wu H Wu C Lu Q Ding Z Xue M Lin J. Spatial-temporal characteristics of severe fever with thrombocytopenia syndrome and the relationship with meteorological factors from 2011 to 2018 in Zhejiang Province, China. PLoS Negl Trop Dis. (2020) 14:e0008186. 10.1371/journal.pntd.0008186 32255791 PMC7164674

[18] Huang X Wang S Wang X Lyu Y Jiang M Chen D. Estimation of the incidence of severe fever with thrombocytopenia syndrome in high endemic areas in China: an inpatient-based retrospective study. BMC Infect Dis. (2018) 18:66. 10.1186/s12879-018-2970-7 29402229 PMC5800001

[19] Silvas JA Aguilar PV. The emergence of severe fever with thrombocytopenia syndrome virus. Am J Trop Med Hyg. (2017) 97:992–6. 10.4269/ajtmh.16-0967 28820686 PMC5637595

[20] Sun JM Zhang YJ Gong ZY Zhang L Lv HK Lin JF. Seroprevalence of severe fever with thrombocytopenia syndrome virus in southeastern China and analysis of risk factors. Epidemiol. Infect. (2015) 143:851–6. 10.1017/S0950268814001319 24866248 PMC4411641

[21] Cliff AD Ord JK. Spatial Processes: Models & Applications. London: Taylor & Francis (1981).

[22] Getis A Ord JK. The analysis of spatial association by use of distance statistics. In: Perspectives on Spatial Data Analysis. Berlin; Heidelberg: Springer (2010). p. 127–45. 10.1007/978-3-642-01976-0_10

[23] Ord JK Getis A. Testing for local spatial autocorrelation in the presence of global autocorrelation. J Regional Sci. (2001) 41:411–32. 10.1111/0022-4146.00224

[24] Wang Y Li K Li P Sun J Ye L Dai Y. Community-based comprehensive measures to prevent severe fever with thrombocytopenia syndrome, China. Int J Infect Dis. (2018) 73:63–6. 10.1016/j.ijid.2018.06.002 29894732

[25] Chen Z Yang X Bu F Yang X Yang X Liu J. Ticks (acari: ixodoidea: argasidae, ixodidae) of China. Exp Appl Acarol. (2010) 51:393–404. 10.1007/s10493-010-9335-2 20101443

[26] Fang LQ Liu K Li XL Liang S Yang Y Yao HW. Emerging tick-borne infections in mainland China: an increasing public health threat. Lancet Infect Dis. (2015) 15:1467–79. 10.1016/S1473-3099(15)00177-2 26453241 PMC4870934

[27] Estrada-Peña A de la Fuente J. The ecology of ticks and epidemiology of tick-borne viral diseases. Antiviral Res. (2014) 108:104–28. 10.1016/j.antiviral.2014.05.016 24925264

[28] Ma X Shao G Zhu G Xu R. Analysis on the investigation results of tick on animals in Ningbo. Chinese J Heal Lab Technol. (2012) 22:1416–7.

[29] Wu XB Na RH Wei SS Zhu JS Peng HJ. Distribution of tick-borne diseases in China. Parasit Vectors. (2013) 6:119. 10.1186/1756-3305-6-119 23617899 PMC3640964

