# Supplementary material for: Severe Fever With Thrombocytopenia Syndrome in Southeastern China, 2011–2019
Source: Front Public Health. 2022 Feb 9;9:803660. doi: 10.3389/fpubh.2021.803660 (PMC8864090; doi:10.3389/fpubh.2021.803660)
Supplement: Supplementary file 1 [file Table_1.DOCX]

***Supplementary Material***

# Supplementary Figures and Tables

## Supplementary Tables

Table 1. The cities of Zhejiang province and their subordinate counties

| **City** |  | Subordinate Counties | | | | | | | | | | | | |
| --- | --- | --- | --- | --- | --- | --- | --- | --- | --- | --- | --- | --- | --- | --- |
| **Hangzhou** | Shangcheng | | Xiacheng | Jiangan | Gongshu | Xihu | Binjiang | Xiaoshan | Yuhang | Tonglu | Chun'an | Jiande | Fuyang | Lin'an |
| **Ningbo** |  | Haishu | Jiangbei | Beilun | Zhenhai | Yinzhou | Xiangshan | Ninghai | Yuyao | Cixi | Fenghua | |  |  |
| **Wenzhou** |  | Lucheng | Longwan | Ouhai | Dongtou | Yongjia | Pingyang | Cangnan | Wencheng | Taishun | Rui'an | Yueqing | |  |
| **Jiaxing** |  | Nanhu | Xiuzhou | Jiashan | Haiyan | Haining | Pinghu | Tongxiang | |  |  |  |  |  |
| **Huzhou** |  | Wuxing | Nanxue | Deqing | Changxing | Anji |  |  |  |  |  |  |  |  |
| **Shaoxing** |  | Yuecheng | Keqiao | Xinchang | Zhuji | Shangyu | Shengzhou | |  |  |  |  |  |  |
| **Jinhua** |  | Wucheng | Jindong | Wuyi | Pujiang | Pan'an | Lanxi | Yiwu | Dongyang | Yongkang | |  |  |  |
| **Quzhou** |  | Kecheng | Qujiang | Changshan | Kaihua | Longyou | Jiangshan | |  |  |  |  |  |  |
| **Zhoushan** |  | Dinghai | Putuo | Daishan | Shengsi |  |  |  |  |  |  |  |  |  |
| **Taizhou** |  | Jiaojiang | Huangyan | Luqiao | Yuhuan | Sanmen | Tiantai | Xianju | Wenling | Linhai |  |  |  |  |
| **Lishui** |  | Liandu | Qingtian | Jinyun | Suichang | Songyang | Yunhe | Qingyuan | Longquan | Jingning Shezu Zizhi | | |  |  |

Table 2. The epidemiological characteristics of SFTS cases during 2011–2019

| Characteristics | 2011 | 2012 | 2013 | 2014 | 2015 | 2016 | 2017 | 2018 | 2019 | Total |
| --- | --- | --- | --- | --- | --- | --- | --- | --- | --- | --- |
|  | n= 9 | n= 25 | n= 31 | n= 57 | n= 72 | n= 66 | n= 71 | n= 84 | n= 48 | n= 463 |
| Outcome |  |  |  |  |  |  |  |  |  |  |
| Survivors | 9 | 22 | 27 | 47 | 64 | 60 | 63 | 74 | 44 | 410 |
| Deaths | 0 | 3 | 4 | 10 | 8 | 6 | 8 | 10 | 4 | 53 |
| CFR | 0.00% | 12.00% | 12.90% | 17.54% | 11.11% | 9.09% | 11.27% | 11.90% | 8.33% | 11.45% |
| Gender |  |  |  |  |  |  |  |  |  |  |
| Male | 3 | 12 | 19 | 24 | 22 | 36 | 37 | 46 | 24 | 223 |
| Female | 6 | 13 | 12 | 33 | 50 | 30 | 34 | 38 | 24 | 240 |
| M-to-F ratio | 0.50 | 0.92 | 1.58 | 0.73 | 0.44 | 1.20 | 1.09 | 1.21 | 1.00 | 0.93 |
| Occupation (N, %) | |  |  |  |  |  |  |  |  |  |
| Farmer | 3(33.3) | 15(60.0) | 17(54.8) | 32(56.1) | 48(66.7) | 47(71.2) | 56(78.9) | 64(76.2) | 39(76.2) | 321(69.3) |
| Household | 5(55.6) | 8(32.0) | 9(29.0) | 15(26.3) | 21(29.2) | 17(25.8) | 10(14.1) | 9(10.7) | 4(10.7) | 98(21.2) |
| Other | 1(11.1) | 2(8.0) | 5(16.1) | 10(17.5) | 3(4.2) | 2(3.0) | 5(7.0) | 11(13.1) | 5(13.1) | 44(9.5) |
| Age (years) |  |  |  |  |  |  |  |  |  |  |
| Median | 70.00 | 70.00 | 63.00 | 64.00 | 65.00 | 67.00 | 68.00 | 65.00 | 65.50 | 66.00 |
| Q1 | 62.50 | 56.50 | 56.00 | 51.00 | 57.00 | 56.00 | 60.00 | 56.25 | 56.50 | 57.00 |
| Q3 | 74.00 | 77.00 | 75.00 | 69.50 | 75.00 | 76.50 | 75.00 | 72.00 | 73.75 | 74.00 |
| Illness onset to diagnosis | |  |  |  |  |  |  |  |  |  |
| Median | 12.00 | 14.00 | 7.00 | 7.00 | 5.00 | 4.00 | 5.00 | 5.00 | 4.00 | 6.00 |
| Q1 | 9.00 | 8.00 | 4.00 | 5.00 | 3.00 | 2.00 | 3.00 | 3.00 | 2.00 | 3.00 |
| Q3 | 16.00 | 23.00 | 10.00 | 9.50 | 8.00 | 6.25 | 8.00 | 8.00 | 8.00 | 9.00 |
